# Surgical Site Infections in Breast Surgery: The Use of Preoperative Antibiotics for Elective, Nonreconstructive Procedures

**DOI:** 10.1155/2016/1645192

**Published:** 2016-10-05

**Authors:** Christopher B. Crawford, James A. Clay, Anna S. Seydel, Jessica A. Wernberg

**Affiliations:** ^1^Department of Surgery, University of Nebraska Medical Center, Omaha, NE, USA; ^2^Department of General Surgery, Marshfield Clinic, Marshfield, WI, USA

## Abstract

*Background*. Antibiotic prophylaxis for surgical site infections (SSIs) for breast surgery is widespread, but the benefit in clean surgical cases is not well defined.* Methods*. A retrospective analysis of 855 patients undergoing elective, nonreconstructive breast operations was performed, with 401 patients receiving no antibiotics and 454 patients receiving a single dose of preoperative antibiotic.* Results*. Administration of a preoperative antibiotic did not decrease the SSI rate. In this community-based study, antibiotic use practices varied considerably by surgeon. In univariate analyses, SSI rates appeared to increase with prophylactic antibiotic use (12% SSI with antibiotics versus 4% without, *p* < 0.0001), likely because the use of underdosed antibiotics was associated with higher rates of SSI (13.2% SSI with cefazolin 1 gram, *p* < 0.0001, and 15.4% SSI with clindamycin 300 mg or less, *p* = 0.0269). Methicillin-resistant* Staphylococcus aureus* was the most common isolate from SSI cultures, 31.8% (7 of 22). In multivariable analyses, increased risk of SSI was associated with BMI > 25 kg/m^2^ (OR: 1.08, 95% CI: 1.04–1.11, *p* < 0.0001).* Conclusion*. The administration of a single dose of preoperative antibiotic did not decrease the rate of SSI in this large series of patients undergoing clean breast operations. BMI >25 kg/m^2^ and the use of an inadequate dose of antibiotics for prophylaxis may increase risk of SSI.

## 1. Introduction

Surgical site infection (SSI) is a commonly reported source of postsurgical morbidity. In breast surgery, three separate reviews of the National Surgical Quality Improvement Program (NSQIP) database demonstrated SSI rates of 1.4%–3.2% [1–3]. Despite the relatively low SSI rates in registry data, other studies have reported SSI rates of up to 36% for procedures such as modified radical mastectomy [4]. Additional randomized controlled trials (RCTs) reported SSI rates ranging from 3.2% to 18.9% [5–11]. The historically cited rate for clean surgical cases is 1.5% [12]. When an SSI occurs, it can impact patient recovery and result in added cost and hospital readmission. To alleviate these concerns, perioperative antibiotics have been used in an attempt to decrease the rate of SSI in breast surgery for both benign and malignant indications.

A large RCT published by Hall et al. in 2006 evaluated the use of antibiotics in nonreconstructive breast surgery for both benign and malignant pathologies. It failed to show a difference in SSI rates with the use of a preoperative antibiotic not available in the USA, flucloxacillin [5]. In contrast, a 2014 Cochrane Collaboration review demonstrated a beneficial effect of preoperative antibiotics in breast cancer surgery [13]. The question of whether preoperative antibiotic use decreases SSI using antibiotics available in the USA in a comprehensive breast surgery practice remains unanswered. Thus, we sought to investigate the impact of a timely single dose of preoperative antibiotic on SSI rates in an elective, nonreconstructive breast surgery population encompassing both cancer and cosmetic operations.

## 2. Methods

A retrospective records analysis was performed for patients who underwent elective, nonreconstructive breast surgery between 2008 and 2012 at the National Accreditation Program for Breast Centers (NAPBC) accredited institutions: the Marshfield Clinic Ambulatory Surgery Center or Ministry Saint Joseph's Hospital, a tertiary referral hospital in Marshfield, Wisconsin. Marshfield Clinic Institutional Review Board approval was obtained. Current Procedural Terminology (CPT) codes were used to identify patients who had elective breast surgery not involving simultaneous placement of an implant or tissue expander. The procedures were performed by surgical oncologists or plastic surgeons. Operative indications included benign and malignant disease. Patients undergoing a concurrent sentinel lymph node biopsy or lymphadenectomy were included. Selection of antibiotic was at the discretion of the surgeon.

Procedures were classified as small and large surface area because procedures involving larger surface areas alone have been shown to increase the risk of SSI [2]. Small surface area procedures included partial mastectomy, breast biopsy, and sentinel lymph node biopsy. The CPT codes for small surface area procedures included 19101, 19110, 19120, 19125, 19126, and 19301. Large surface area procedures included mastectomy, axillary lymph node dissection, reduction mammaplasty, and mastopexy. The CPT codes for large surface area procedures included 19300, 19302, 19303, 19305, 19307, 19316, and 19318. Preoperative placement of a localizing wire was documented. Reduction mammaplasty and mastopexy were captured as a separate variable.

Exclusion criteria included placement of a breast implant or tissue expander, evidence of preoperative infection, improper timing of antibiotic administration (administration after incision or more than 1 hour earlier or timing not clearly recorded), treatment with an antibiotic within 30 days for an unrelated infectious process, a concurrent major operation at a secondary site, or absence of surgical follow-up within 30 days.

Data was gathered by clinical chart review. Initial abstraction was done by the primary author and verified by the second. The operative reports were reviewed to ensure that the procedures were properly coded. Patients who underwent bilateral procedures were considered as a single subject. If one side of a bilateral procedure was a large surface area procedure, the patient was included in the large surface area analysis. Timing of antibiotic administration, dose, and time of incision were gathered from perioperative nursing, pharmacy, and anesthesia records. All Marshfield Clinic and Saint Joseph's Hospital documentation within at least 30 days of the procedure was reviewed. The diagnosis of an SSI was documented by the surgeon of record based on postoperative clinical examination and/or the presence of positive bacterial cultures from wound drainage within 30 days of the procedure.

## 3. Statistical Analysis

Assuming a 5% absolute difference in SSI rates between patients who received a single preoperative dose of antibiotic and those who did not, a minimum of 400 cases and 400 controls were necessary to attain 80% statistical power. This was based on an expected difference of SSI rates from 8% for the patients who did not receive any antibiotics to 3% for those who received a single dose of preoperative antibiotic [6, 7, 9–11, 14–16]. In the bivariable analysis, a *Z*-/*t*-test was used for normally distributed data, Wilcoxon Rank-Sum test for skewed data, and Fisher's Exact or Chi-square test for categorical data. Controls were chosen as the referent group in order to derive the odds ratio, 95% confidence interval, and corresponding *p* value for antibiotic use in association with the SSI outcome. Similarly, analyses were performed for the following clinical variables: wire localization, body mass index (BMI), diabetes mellitus, sex, prior operation within 30 days, surgeon, small or large surface area, BMI > 25 or ≤ 25 kg/m^2^, and age > 50 or ≤ 50 years, as well as whether the procedure was a reduction mammaplasty or mastopexy. Age and BMI were assessed as both binary and continuous variables. A cross-tab analysis comparing antibiotic use and resulting SSI rate was conducted to determine whether certain subgroups of patients benefited from preoperative antibiotic use. These groups were stratified by age > 50 or ≤ 50 years, sex, BMI > 25 or ≤ 25 kg/m^2^, large surface area, small surface area, diabetes, wire localization, previous ipsilateral breast operation within 30 days, and whether or not the operation was a reduction mammaplasty or mastopexy. Antibiotic dosages were also analyzed for significant associations with SSI between patients with similar BMI.

In the multivariable analysis, stepwise logistic regression modeling was applied to determine the set of statistically significant clinical risk factors in association with the SSI outcome. A *p* value of <0.05 is considered statistically significant. All data analyses were carried out using the commercially available statistical software package, SAS, version 9.3, English (Cary, NC).

## 4. Results

Records of 1,461 patients were reviewed. Of these patients, 606 were excluded: 120 received implants or expanders within 30 days, 6 had a documented breast infection prior to operation, 145 had improper timing of antibiotics, 249 were given postoperative antibiotics prophylactically or for unrelated conditions, and 18 had no follow-up within 30 days, leaving 855 qualifying patients. The study contained 454 patients who received a single dose of preoperative prophylactic antibiotic within 60 minutes of incision and 401 patients who did not receive antibiotics.

Patient demographics and rate of antibiotic administration are displayed in Table 1. Antibiotics were used differently among subgroups. Patients with large surface area procedures were more likely to receive an antibiotic. Patients with a localizing wire were less likely to receive an antibiotic. Providers used antibiotics with different frequencies and dosages. Using age and BMI as continuous variables, younger patients and obese patients were more likely to receive antibiotics.

Table 2 displays the main outcomes by clinical characteristics of the patient population. The overall surgical site infection rate was 8.3% (71 of 855 patients). The SSI rate for patients who received a dose of preoperative antibiotic was 12.1% versus 4% for those who did not (*p* value < 0.0001). There was an increase in SSI rates for patients with a previous ipsilateral breast operation within 30 days, a large surface area procedure, or a BMI > 25 kg/m^2^. Elevated BMI was associated with an increased SSI rate. Reduction mammaplasty and mastopexy were independently associated with a higher SSI rate. Localizing wire placement was associated with a lower SSI rate.

Twenty-two of the 71 patients with a surgical site infection had cultures at the time of clinical diagnosis. The most common bacterium cultured was methicillin-resistant* Staphylococcus aureus* (MRSA), 31.8% (7 of 22). This was followed by coagulase negative* Staphylococcus*, methicillin-susceptible* Staphylococcus aureus* (MSSA), and* Escherichia coli*: 22.7%, 13.6%, and 9.1%, respectively. Five patients had no growth from their cultures but were still felt to have an SSI clinically.

**Figure 1:**
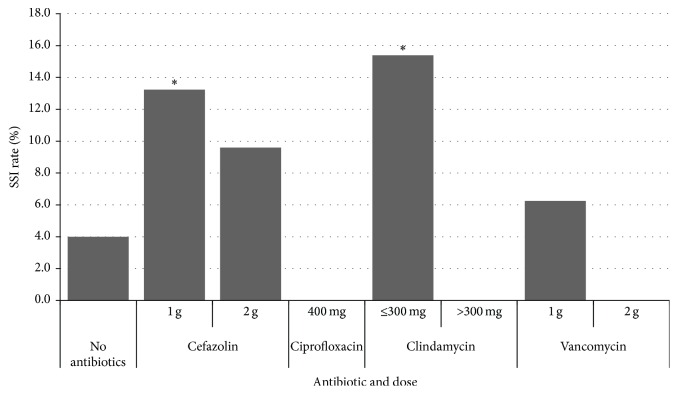
Surgical site infection rate by antibiotic and dose. Rates of SSI in nonreconstructive breast surgery patients were compared by antibiotic and dose. Clindamycin doses^∗^ of 150 or 300 mg were combined as ≤300 mg and doses of 450 and 600 mg were combined as >300 mg. The 1-gram cefazolin dose was associated with significantly higher rates of SSI compared to no antibiotic prophylaxis, 13.2% versus 4.0%, *p* < 0.0001. A ≤300 mg dose of clindamycin was also associated with significantly higher rates of SSI compared to no antibiotics, 15.4% versus 4.0%, *p* = 0.027.

The antibiotic agent used in 87.7% of cases was cefazolin. The remaining patients received clindamycin (7.3%), vancomycin (3.7%), or ciprofloxacin (1.3%). Underdosing of antibiotics was common in those patients who did receive antibiotics. Of those who were treated with cefazolin, 325 (81%) received only 1 gram and none of the patients who weighed ≥120 kg received the currently recommended 3 grams [17]. In fact, patients who received 1 gram of cefazolin developed SSI at a significantly higher rate than those not receiving antibiotics (13.2% versus 4.0%, *p* < 0.0001, OR: 3.7, 95% CI: 2.03–6.65) (Figure 1). Similarly, the 26 patients who received 300 mg or less of clindamycin had significantly higher rates of infection compared to no antibiotic prophylaxis (15.4% versus 4.0%, *p* = 0.03, OR: 4.4, 95% CI: 1.3–14.2). In contrast, patients who received antibiotic prophylaxis with 2 grams of cefazolin or more than 300 mg of clindamycin did not have a significantly different SSI rate from those that did not have prophylactic antibiotics.

To compare antibiotic use and SSI rate in subgroups, a cross-tab analysis was performed (Table 3). The subsets that attained significance in the cross-tab analysis were no prior operation within 30 days, female sex, small surface area procedure, placement of a localizing wire, BMI > 25 kg/m^2^, and a procedure other than reduction mammaplasty or mastopexy. In all of these groups, use of an antibiotic was associated with an increased SSI rate.

To better understand the effect of antibiotics on SSI, a multivariable stepwise logistic regression analysis was performed using the following variables: antibiotic use, diabetes, sex, prior operation, surgeon, small or large surface area, BMI > or ≤ 25 kg/m^2^, age > 50 or ≤ 50 years, and patients with reduction mammaplasty or mastopexy (Table 4). In the multivariable analysis, antibiotics use did not affect the rate of SSI. The only variables that maintained significance were localizing wire placement and BMI. A localizing wire was associated with a decreased SSI rate (odds ratio [OR]: 0.17, 95% confidence interval [CI]: 0.08–0.36, *p* < 0.0001) and BMI >25 kg/m^2^ was associated with an increased SSI rate (OR: 1.08, 95% CI: 1.04–1.11, *p* < 0.0001).

## 5. Discussion

Modern healthcare is accompanied by increased monitoring of individual and institutional outcomes. Pay-for-performance reimbursement models and CMS “never events” take into consideration postoperative complications. This affects all surgeons that operate on the breast for benign or malignant indications, not to mention the wellbeing of their patients. In some disciplines, SSI rates can be reduced with a single dose of preoperative antibiotic and a recent Cochrane review suggests that this may be the case in the context of breast cancer surgery [3]. However, in this retrospective observational study of elective, nonreconstructive breast operations, a single dose of preoperative antibiotic was not associated with a lower SSI rate. In fact, inadequately dosed antibiotic prophylaxis seemed to increase the rate of SSI. However, logistical regression analysis found BMI to be the key variable associated with increased SSI while wire localization decreased the rate of SSI.

Three separate NSQIP database reviews showed that the SSI rates in breast surgery range from 1.4% to 3.2% [1–3], suggesting that SSI rates may be decreasing compared to previous RCTs where reported SSI rates range from 3 to 19% [6–11]. This decrease could be due to the implementation of Surgical Care Improvement Project (SCIP) measures, improved skin preparation agents, or lower rates of SSI in breast-conserving surgery [2, 4, 18]. The NSQIP reviews may also be underreporting the SSI rates in breast surgery, as the overall complication rates are lower in the NSQIP data compared to single-institution studies [2]. This may be the case in this study, where the observed rate of SSI following breast surgery was 8.3% overall. Lower SSI rates in registry data reviews compared to single-institution studies have been reported in other surgical subspecialties, such as vascular surgery [19]. The largest review of the NSQIP database examined only female patients, and bilateral mastectomy was regarded as two procedures, thus increasing the number of cases without increasing the number of patients [3]. The impact this had on reported SSI rates is unclear, but it could potentially decrease the overall SSI rate. The NSQIP database also does not include information on the administration or timing of antibiotics; thus, the SSI rates based on these data cannot be directly compared to SSI rates in RCTs looking at antibiotic use. Our observation is that timely administration of preoperative antibiotics to a patient undergoing breast surgery does not play an important role in the reduction of surgical site infections.

In fact, the use of prophylactic antibiotics with dosages below current recommended dosing guidelines may increase the risk of SSI as seen in our study (Figure 1) [17]. This parallels the results of Olsen et al., who found that suboptimal prophylactic antibiotic dosing was a significant independent risk factor for SSI in major breast surgery [20]. The operations included in our study were done prior to recent antibiotic prophylaxis recommendations for higher doses, and our results suggest that antibiotics dosed based on current recommendations do not significantly alter the rate of SSI when compared to no antibiotic prophylaxis.

Staphylococci species were the most common bacteria isolated from the subset of patients with SSIs that were cultured. This compares similarly to a previous study which found 60% of cultures from SSIs complicating breast surgery isolated staphylococci species [21]. Also, in our study, drug resistant variants were common, with MRSA isolated in 31.8% of cultures. This corresponds to 9.8% of all the SSIs being MRSA infections; however, this likely underestimates the true number given that only 31% of the clinically diagnosed SSIs were cultured. This observation supports the use of cultures with susceptibility profiles in patients with SSI following breast surgery as suggested by Throckmorton et al. [21]. In other clean operations with higher than expected rates of MRSA infections, such as vascular surgery, identifying preoperative patient risk factors for MRSA and the use of prophylactic antibiotics able to cover MRSA had been advocated for [22]. Further investigation into whether this would benefit breast surgery patients as well may be warranted.

Another finding in the study was that preoperative localizing wire placement was associated with a lower SSI rate than in procedures with no wire placement. However, preoperative instrumentation has been shown to increase the rate of SSI [23]. Our observation is likely because localizing wire placement was a surrogate marker for surface area, given that 98% (341/348) of the patients who received a localizing wire had small surface area procedures. However, with large surface area procedures such as mastectomy, there would be no need for wire localization.

The other significant risk factor for SSI identified in multivariable analysis was a BMI indicative of being overweight or obese, supporting previous studies [2, 3, 18, 24]. Obese patients were more likely to receive antibiotic prophylaxis. The median BMI of patients who did not receive an antibiotic was 28.3 kg/m^2^. The median BMI of patients who did receive an antibiotic was 30.0 kg/m^2^ (*p* = 0.0003, Table 1). However, despite the increased SSI rate in patients with a BMI > 25 kg/m^2^ (Table 2), the use of an antibiotic did not decrease this rate. Obese patients have thicker adipose layers, increasing operative surface area. The increased dead space and poor perfusion of fatty tissue increase the risk of local wound infection. Additionally, obesity has substantial effects on the immune system with impaired chemotaxis, dysregulated immune response, and altered macrophage differentiation [25]. Additionally, antibiotic dosing can be challenging in obese patients. Physicians frequently inaccurately dose antimicrobials because obesity affects volume of distribution (*V*_*d*_) of drugs, increasing *V*_*d*_ of lipophilic drugs and decreasing *V*_*d*_ of hydrophilic drugs [26].

Studies evaluating the use of antibiotics in reduction mammaplasty or mastopexy have conflicting results as to the role of antibiotics in preventing SSI [6, 16, 27]. In this study, patients undergoing large surface area procedure were more likely to receive an antibiotic (81%, *p* < 0.0001), particularly if it was a reduction mammaplasty or mastopexy (98%, *p* < 0.0001). There were increased SSI rates in both large surface area (13%, *p* < 0.0001) and reduction mammaplasty and mastopexy procedures (14%, *p* = 0.0003, Table 2), but the use of an antibiotic did not decrease SSI in either group (Table 3).

Inherent limitations with our study include its retrospective design, variability in the patient population among surgeons, and variability in the rates of antibiotic administration among patient groups. These introduce the potential for selection bias.

The classification of large surface area and small surface area procedures is subjective. A woman with large breasts undergoing a lumpectomy may actually have an operation involving more surface area than a woman with small breasts undergoing a mastectomy. The results must be interpreted with this limitation in mind. Volume of resection or specimen weight would likely be a more accurate measure but may not be known preoperatively during the decision of prophylactic antibiotic use or not.

Additionally, in an attempt to make our study generalizable, we included both benign and malignant disease. Malignancy has been associated with immunosuppression and Angarita et al. found advanced tumors of the breast associated with increased SSI [18]. We did not see a difference in the rate of SSI following operations for cancer versus benign indications (data not shown). Because this study was designed to assess the impact of timely prophylactic antibiotics on a broad breast surgery patient population and not the effect of tumors on immunosuppression, detailed analysis of the cancer subset was not undertaken which is another limitation of the study. Future research targeting antibiotic prophylaxis in different subsets of breast surgery patients may be required to identify those patients that would benefit the most.

Again, in an attempt to have general applicability, both male and female patients were included. Obviously, far less data is available on male breast operations and there is the possibility of a gender difference in SSI. However, a small number of male patients included in our study were nearly equally divided between receiving antibiotics and not and none developed an SSI, so including them did not change the main findings of the study.

## 6. Conclusions

Given the increased surgical site infection rate in breast surgery compared to clean cases overall, our study aimed to determine whether the SSI rate was decreased by prophylactic antibiotic administration in a community-based clinical practice. While certain patient groups are more prone to developing SSI, such as those with elevated BMI, we found that a timely single dose of preoperative antibiotic did not lower the SSI rate overall or in at-risk groups. Inadequately dosed antibiotics may actually increase the risk of SSI. Our data suggests that when prescribed, antibiotics should at least follow current dosing recommendations. Of note, the most common organism identified in our SSIs was MRSA, which lends favor to the practice of obtaining cultures with susceptibilities in suspected postoperative wound infections following breast surgery. We also observed significant variability in antibiotic prescribing practices from surgeon to surgeon. Despite the perceived low cost and relative low morbidity, antibiotics without benefit should not be prescribed. This study, which included both benign and malignant conditions, challenges the routine practice of preoperative antibiotic use for elective, nonreconstructive breast surgery.

## Figures and Tables

**Table 1:** Clinical characteristics of patients in relation to antibiotic administration.

	Antibiotic given		*p* value^†^
	No	Yes	
	*n* = 401	*n* = 454	
*Sex*			**0.5029**
Female	390 (97%)	445 (98%)	
Male	11 (3%)	9 (2%)	
*Diabetes*			**0.3243**
No	352 (88%)	409 (90%)	
Yes	49 (12%)	45 (10%)	
*Wire localization*			**<0.0001**
No	143 (36%)	364 (80%)	
Yes	258 (64%)	90 (20%)	
*Surface area*			**<0.0001**
Small surface area	324 (81%)	119 (26%)	
Large surface area	77 (19%)	335 (74%)	
*Surgeon*			**<0.0001**
A (surgical oncology)	65 (16%)	108 (24%)	
B (plastic surgery)	3 (1%)	188 (41%)	
C (plastic surgery)	7 (2%)	79 (17%)	
D (plastic surgery)	5 (1%)	0 (0%)	
E (surgical oncology)	321 (80%)	79 (17%)	
*Mastopexy or reduction mammaplasty*			**<0.0001**
No	395 (99%)	196 (43%)	
Yes	6 (1%)	258 (57%)	
*Age, median (IQR)*	59 years (72–49)	49 years (64–36)	**<0.0001** ^‡^
*BMI, median (IQR)*	28.3 kg/m^2^ (33.6–24.4)	30.0 kg/m^2^ (35.2–26.1)	**0.0003** ^‡^

^†^ *p* value was derived from Fisher's Exact test.

^‡^ *p* value was derived from Wilcoxon Rank-Sum test.

IQR: interquartile range.

**Table 2:** Clinical characteristics and outcomes of the patient population.

	No infection	Surgical site infection	*p* value^†^
	*n* = 784	*n* = 71	
*Single dose of antibiotic given*			**<0.0001**
No	385 (49%)	16 (23%)	
Yes	399 (51%)	55 (77%)	
*Sex*			**0.4000**
Female	764 (97%)	71 (100%)	
Male	20 (3%)	0 (0%)	
*Age (years)*			**0.5329**
>50	451 (58%)	38 (54%)	
≤50	333 (42%)	33 (46%)	
*Body mass index (BMI)*			**0.0274**
>25	593 (76%)	62 (87%)	
≤25	191 (24%)	9 (13%)	
*Diabetes *			**0.2316**
No	701 (89%)	60 (85%)	
Yes	83 (11%)	11 (15%)	
*Wire localization*			**<0.0001**
No	444 (57%)	63 (89%)	
Yes	340 (43%)	8 (11%)	
*Surface area*			**<0.0001**
Small surface area	427 (54%)	16 (23%)	
Large surface area	357 (46%)	55 (77%)	
*Mastopexy or reduction mammaplasty*			**0.0003**
No	556 (71%)	35 (49%)	
Yes	228 (29%)	36 (51%)	
*Age, median (IQR)*	54 years (68–43)	52 years (62–44)	**0.5299** ^‡^
*BMI, median (IQR)*	28.9 kg/m^2^ (34.0–25.1)	33.3 kg/m^2^ (38.5–29.6)	**<0.0001** ^‡^

^†^ *p* value was derived from Fisher's Exact test.

^‡^ *p* value was derived from Wilcoxon Rank-Sum test.

IQR: interquartile range.

**Table 3:** Cross-tab analysis of clinical characteristics and antibiotic use in relation to SSI rate.

	No infection	Surgical site infection	*p* value^†^
	*n* = 784	*n* = 71	
*No prior operation within 30 days*			<0.0001
No antibiotics	366 (47%)	13 (18%)	
Single dose of antibiotics	386 (49%)	51 (72%)	
*Prior operation within 30 days*			0.6766
No antibiotics	19 (2%)	3 (4%)	
Single dose of antibiotics	13 (2%)	4 (6%)	

*Male patients*			NA^*∗*^
No antibiotics	11 (1%)	0 (0%)	
Single dose of antibiotics	9 (1%)	0 (0%)	
*Female patients*			<0.0001
No antibiotics	374 (48%)	16 (23%)	
Single dose of antibiotics	390 (50%)	55 (77%)	

*Small surface area*			0.0170
No antibiotics	317 (40%)	7 (10%)	
Single dose of antibiotics	110 (14%)	9 (13%)	
*Large surface area*			0.7136
No antibiotics	68 (9%)	9 (13%)	
Single dose of antibiotics	289 (37%)	46 (65%)	

*No wire localization*			0.1791
No antibiotics	130 (17%)	13 (18%)	
Single dose of antibiotics	314 (40%)	50 (70%)	
*Wire localization placed*			0.0299
No antibiotics	255 (33%)	3 (4%)	
Single dose of antibiotics	85 (11%)	5 (7%)	

*Nondiabetic patients*			0.0002
No antibiotics	338 (43%)	14 (20%)	
Single dose of antibiotics	363 (46%)	46 (65%)	
*Diabetic patients*			0.0233
No antibiotics	47 (6%)	2 (3%)	
Single dose of antibiotics	36 (5%)	9 (13%)	

*BMI ≤ 25*			0.1904
No antibiotics	108 (14%)	3 (4%)	
Single dose of antibiotics	83 (11%)	6 (9%)	
*BMI > 25*			<0.0001
No antibiotics	277 (35%)	13 (18%)	
Single dose of antibiotics	316 (40%)	49 (69%)	

*Surgery age ≤ 50*			0.0009
No antibiotics	125 (16%)	3 (4%)	
Single dose of antibiotics	208 (27%)	30 (42%)	
*Surgery age > 50*			0.0062
No antibiotics	260 (33%)	13 (18%)	
Single dose of antibiotics	191 (24%)	25 (35%)	

*Any breast procedure not including mastopexy or reduction mammaplasty*			0.0089
No antibiotics	379 (48%)	16 (23%)	
Single dose of antibiotics	177 (23%)	19 (27%)	
*Mastopexy or reduction mammaplasty*			1.0000
No antibiotics	6 (1%)	0 (0%)	
Single dose of antibiotics	222 (28%)	36 (51%)	

^†^ *p* value was derived from Fisher's Exact test.

^*∗*^Not applicable.

**Table 4:** Multivariable stepwise logistic regression modeling for risk factors^1^ in association with infection.

	Regression coefficient (*R*)	Odds ratio (OR)	95% confidence interval (CI)	*p* value
*Wire localization*				
No^2^		1.00		
Yes	−1.7695	0.17	0.08–0.36	<0.0001
*BMI (kg/m* ^2^)	0.0741	1.08	1.04–1.11	<0.0001

^1^The following risk factors were also included in the stepwise logistic regression modeling selection: antibiotic use, diabetes, gender, prior surgery, surgeon, large surface area, BMI > 25 kg/m^2^ (yes/no), surgery age, surgery age > 50 years (yes/no), and mammoplasty.

^2^Referent group.
